# Genomic and Phenomic Screens for Flower Related RING Type Ubiquitin E3 Ligases in *Arabidopsis*

**DOI:** 10.3389/fpls.2017.00416

**Published:** 2017-03-28

**Authors:** Mirko Pavicic, Katriina Mouhu, Feng Wang, Marcelina Bilicka, Erik Chovanček, Kristiina Himanen

**Affiliations:** ^1^Department of Agricultural Sciences, University of HelsinkiHelsinki, Finland; ^2^Viikki Plant Science Centre, University of HelsinkiHelsinki, Finland

**Keywords:** *Arabidopsis*, flower, RING E3 ligase, ubiquitin, high throughput, image based phenotyping, phenomics data analysis

## Abstract

Flowering time control integrates endogenous as well as environmental signals to promote flower development. The pathways and molecular networks involved are complex and integrate many modes of signal transduction. In plants ubiquitin mediated protein degradation pathway has been proposed to be as important mode of signaling as phosphorylation and transcription. To systematically study the role of ubiquitin signaling in the molecular regulation of flowering we have taken a genomic approach to identify flower related Ubiquitin Proteasome System components. As a large and versatile gene family the RING type ubiquitin E3 ligases were chosen as targets of the genomic screen. The complete list of *Arabidopsis* RING E3 ligases were retrieved and verified in the *Arabidopsis* genome v11 and their differential expression was used for their categorization into flower organs or developmental stages. Known regulators of flowering time or floral organ development were identified in these categories through literature search and representative mutants for each category were purchased for functional characterization by growth and morphological phenotyping. To this end, a workflow was developed for high throughput phenotypic screening of growth, morphology and flowering of nearly a thousand *Arabidopsis* plants in one experimental round.

## Introduction

Flowering time control is a complex network that integrates many modes of signal transduction promoting transition from vegetative stage to reproduction and ultimately leading to the development of flower organs. The endogenous changes that signal the beginning of flowering are referred as autonomous pathways (Amasino and Michaels, 2010). Multiple studies have established the major role that photoperiod has in flowering (Piñeiro and Jarillo, 2013). Flowering in *Arabidopsis* is strongly promoted in long day (LD) conditions but will also ultimately occur under short day (SD) conditions (Steffen et al., 2014). Under LDs, flower induction is dependent on the expression and protein levels of CONSTANS (CO; Suárez-López et al., 2001). Light controls the *CO* transcription via the circadian clock system, inducing a *CO* mRNA peak during the latter part of the day (Suárez-López et al., 2001). *CO* transcription is repressed by CYCLING DOF FACTORs (CDFs; Fornara et al., 2009). Under LDs, the *CO* mRNA afternoon peak coincides with a blue-light activated complex containing FLAVIN-BINDING, KELCH REPEAT, F-BOX 1 (FKF1) and GIGANTEA (GI), which lead *CO* transcription repressors CDFs to degradation (Sawa et al., 2007; Fornara et al., 2009; Song et al., 2012). Additionally, the FKF1-GI complex also stabilizes CO protein in the afternoon (Sawa et al., 2007; Song et al., 2012). CO protein degradation is promoted by at least two ubiquitin E3 ligases: HIGH EXPRESSION OF OSMOTICALLY RESPONSIVE GENES 1 (HOS1) and CONSTITUTIVE PHOTOMORPHOGENIC 1 (COP1; Jang et al., 2008; Lazaro et al., 2012). In the morning, red light promotes HOS1 interaction with CO via phytochrome B activation (Lazaro et al., 2012). COP1 mediates CO protein degradation in a complex with SUPPRESSOR OF PHYA-105 (SPA; Laubinger et al., 2006). In the afternoon, blue light inhibits COP1-SPA-mediated CO degradation by activating CRYPTOCHROME 2 (CRY2) interaction with COP1 (Liu et al., 2008). Thus, both *CO* transcription is up-regulated and CO protein stabilized allowing up-regulation of the mobile flowering signal gene *FLOWERING LOCUS T (FT)* expression in the phloem during the afternoon under LDs, but not under SDs (Piñeiro and Jarillo, 2013). Also regulation of flower development is likely to involve Ubiquitin Proteasome System (UPS) components (Vierstra, 2009).

The UPS has emerged as a powerful regulatory mechanism that facilitates irreversible transitions between developmental stages, and responses to environmental stimuli by selectively degrading short-lived regulators, such as transcription factors and receptors (Sadanandom et al., 2012). Genetic analyses in plants have proposed that this pathway plays a vital role in hormone regulation, floral homeostasis, stress responses and pathogen defense; however, very few targets have been identified in plants apart from the hormone signaling components (Santner and Estelle, 2010). In the UPS system, the highly conserved 76-amino acid protein, ubiquitin, acts as a covalent molecular tag to signal target proteins for proteasome mediated degradation. Ubiquitin attachment requires three distinct enzymatic activities: E1, ubiquitin activating enzymes; E2, ubiquitin conjugating enzymes; and E3, ubiquitin ligase enzymes. Moreover, the UPS consists of accompanying proteins that modulate target recognition and degradation (such as RAD23, SPA1), deubiquitinating enzymes (DUB1) and the proteasome (26S and 20S structures). According Vierstra (2009) over 6% of the Arabidopsis proteome is potentially involved in UPS. However, the common strategy for functionally addressing the role of all UPS components is still evolving. The ubiquitin E3 ligases are the most abundant UPS components and mediate the important recognition of the target proteins for ubiquitination (Kosarev et al., 2002; Stone et al., 2005). The E3 ligases found in plants belong to one of four subtypes: single subunit Homology to E6-AP C-Terminus (HECT), U-box and Really Interesting New Gene (RING) or multisubunit cullin-RING ligases (Sadanandom et al., 2012). The RING-type E3 proteins are the most abundant among the single subunit E3 ligases (Kosarev et al., 2002; Stone et al., 2005).

To unravel the role of the RING type ubiquitin E3 ligase protein family, we took a reverse-genetics approach to identify the RING E3 ligases that could be involved in regulation of *Arabidopsis* flowering time and/or flower development. To this end, we first curated the RING E3 protein family, earlier described by Stone et al. (2005), in the most recent *Arabidopsis* genome release. The *Arabidopsis* protein sequences were subjected to InterProScan for protein domain search and the number of ubiquitin E3 ligases containing RING domains was established to be 509. Association of these RING protein encoding genes with *Arabidopsis* flowering and floral organs was done through the Genevestigator transcriptome database (Hruz et al., 2008). To this end, the expression profiles were divided into categories based on their specificity, high expression or enrichment in flower organs and in the developmental stages of *Arabidopsis*. Several already characterized regulators were identified among these genes, such as the anther dehiscence regulating DAF gene family (Peng et al., 2013), flower size regulating DA2 (Xia et al., 2013) and FRG1 involved in flowering time related DNA methylation (Groth et al., 2014). The well-established flowering time regulator *COP1* fell just below the cut off criteria due to its wide expression profile. A representative mutant collection for each category was obtained from NASC stock center. Additional candidates were also selected based on literature. The genotypically verified mutant collection was subjected to systematic morphological and growth analysis using an automated imaging based plant phenotyping facility. After the thorough vegetative assessment, the flowering time parameters such as number of leaves at bolting and days to bolting were recorded together with morphological analysis of the flower structures. The phenotypic assessment indicated lines with altered growth, morphology, or flowering time. Furthermore, one of the lines showed growth defects in sepals and petals.

## Materials and methods

### Bioinformatic screens

To curate putative RING-type ubiquitin E3 ligases in *Arabidopsis thaliana* genome version ARA11, classification made by Kosarev et al. (2002) and Stone et al. (2005) were used. To this end, the whole *Arabidopsis* proteome was downloaded from ARAPORT (https://www.araport.org/downloads/), and screened with InterProScan for protein families and domain architecture. To confirm that the newly identified RING domain containing protein sequences indeed represented ubiquitin E3 ligase type RING domains, InterProScan 5 (v5.16-55.0) Gene3D, SUPERFAMILY, ProSiteProfiles, SMART, Pfam, and ProSitePatterns signatures were used. Most of InterProScan tools use Hidden Markov Models (HMMs) to detect conserved domains along protein sequences. HMMs have been developed for conserved protein domains and they define for the software, which and where critical residues should be located along the analyzed protein sequence. From the protein domain collection, the ubiquitin E3 ligase type RING domains were filtered according to the criteria provided by Kosarev et al. (2002) and Stone et al. (2005) for canonical RING domains. Once the RING domains were identified, they were aligned with Jalview using ProbCons algorithm with two rounds of pre-training. The metal ligand binding residues were manually inspected and corrected, and small misalignments were edited. Sequences that failed to meet the criteria of InterProScan search engines were not considered in this study.

### Transcriptomic database screens

To associate the curated collection of 509 RING type ubiquitin E3 ligases with flowering the Genevestigator gene expression database software was used (Hruz et al., 2008). The experiments AT-00087, AT-00088, AT-00089, and AT-00090 containing developmental expression data of AtGenExpress initiative microarrays were selected for the analysis (Schmid et al., 2005). In the selected experiments, hybridization probes were available for 393 *RING E3* genes out of the 509. From these experiments, the linear expression data was extracted for the developmental stages of developed rosette, bolting, young flower, developed flower, and flower and silique. For flower organs, the gene expression profiles were extracted for categories of shoot apical meristem (SAM), sepal, petal, stamen, and carpels. In these categories, genes were ranked for their at least 2-fold differential expression against the developed rosette. Their relative expression levels were obtained by log_2_(FC) = log_2_(FL) − log_2_(R), where FC is fold of change, FL is flower organ or development stage and R is rosette. The results for each category were sorted by their log_2_(FC) and all genes with log_2_(FC) > 1 were considered as up-regulated.

### Candidate genes selected by literature

For the candidate approach, we used interaction networks from BioGRID (http://thebiogrid.org/) and cross-checked them with flowering pathway genes listed in the Flowering Interactive Database FLOR-ID (Bouché et al., 2016) to identify RING E3 ligases interacting with known flowering time regulators CONSTANS (CO), CONSTITUTIVE PHOTOMORPHOGENIC 1 (COP1), and TARGET OF EARLY ACTIVATION TAGGED (EAT) 2 (TOE2).

### Plant material and growth conditions

For functional characterization of the identified top most differentially expressed genes and for the selected candidates, *Arabidopsis* mutant lines were obtained from the NASC stock center representing CATMA, SAIL, SALK, and GABI-Kat collections (Alonso et al., 2003; Rosso et al., 2003; Schmid et al., 2005; Kleinboelting et al., 2012). Altogether 49 lines were genotyped by combination of segregation analysis and T-DNA PCR with primers listed in Supplemental Table 1. From these, 43 lines represented 30 unique gene accessions (Supplemental Table 1). As a wild type control, Columbia (Col-0) ecotype of *Arabidopsis thaliana* was used.

For genotyping, plants were grown *in vitro* on MS media supplemented with the corresponding selection. For phenotyping, seeds were sown directly on soil with 50% peat and 50% vermiculite. Trays were covered with plastic wrap and cold stratified at +4 °C for three nights, after which they were transferred to the growth chamber (FytoScope, PSI, Czech Rep.). Seven days after stratification (DAS) the seedlings were transferred to their own pots, placed on the analysis trays and sand was added on top of the peat to prevent growth of any green algae. From the full water saturation of the soil, the water content was let to decrease until 70% and was kept at this level through daily weighing and watering. Growth conditions in the *Arabidopsis* growth chambers were 16 h light/8 h darkness and 22 °C. Relative air humidity of the growth chambers was targeted at 60%. The light intensity was set and controlled at 130 μE (MS6610, Mastech, China).

### Genotyping of the mutant lines

Homozygous one locus mutant lines were confirmed by segregation analysis and T-DNA specific PCRs. The PCR primers, T-DNA position and line information were summarized in Supplemental Table 1. The transcript levels of the T-DNA targeted genes were verified by quantitative real-time PCR (qPCR) analysis. The sample material for qPCR was harvested from the tissue indicated by Arabidopsis eFP Browser for each gene expression pattern: if the gene expression pattern indicated at least moderate expression in flower parts during floral development, tips of inflorescences with developing and open flowers were pooled from three to five individual plants. If the expression was in the seeds, young to mature siliques were pooled. If no expression data via eFP was found, new leaves were pooled with developing and open flowers. Three to four biological replications were harvested for each RNA preparation. RNA was extracted using InviTrap® Spin Plant RNA Kit (STRATEC Molecular), complementary DNA was prepared with SuperScript® IV Reverse Transcriptase (Thermo Fisher Scientific), and the qPCRs were performed using Roche Lightcycler® 480 Instrument II (Roche Diagnostics) using LightCycler® 480 SYBR Green I Master (Roche Diagnostics) with primers listed in Supplemental Table 1. Primers were primarily designed to locus downstream of the T-DNA. The fold up values (mutant line against Col-0) were calculated using the 2^−ΔΔCT^ method according to Livak and Schmittgen (2001). Reference genes used in this study were the most stable *Arabidopsis* genes according to Czechowski et al. (2005): *TIP41 LIKE* (AT4G34270, forward: GTGAAAACTGTTGGAGAGAAGCAA, reverse: TCAACTGGATACCCTTT∧CGCA), *AP2M* (AT5G46630, forward: TTGAAAATTGGAGTACCGTACCAA, reverse: TCCCTCGTATACATCTGGCCA) and *PTB1* (AT3G01150, forward: TTGAAGGAGTGGAATCTCACG, reverse: ATGTGCGGAAAGCAGATACC). Significance level of the qPCR were set at 0.1–0.5 FC for knock-down; <0.1 FC for knock-out; and >2 FC for up-regulated (Supplemental Table 1).

### High throughput plant phenotyping platform

**Figure 1:**
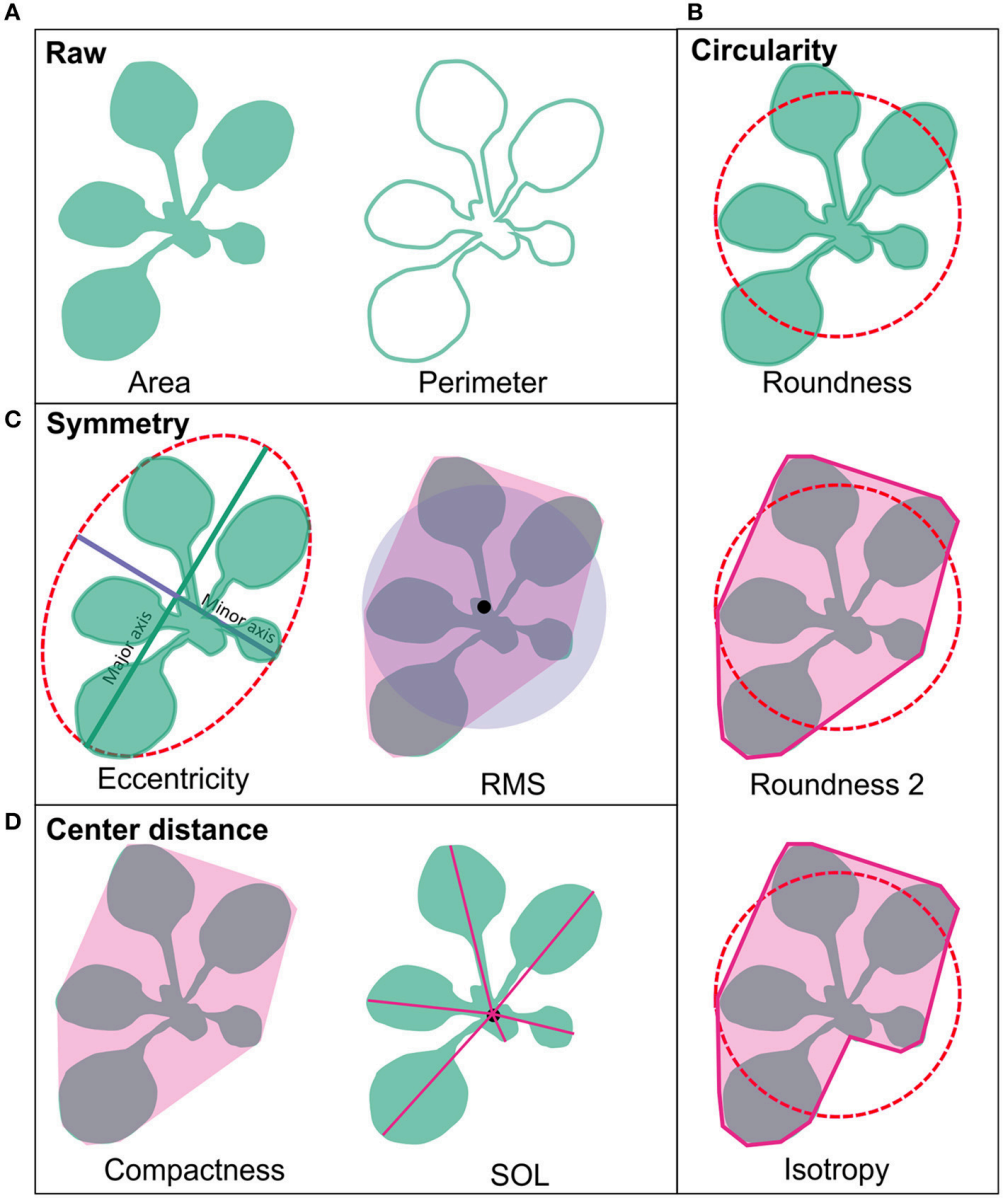
**Rosette morphology parameters. (A)** Area and perimeter of the rosette are raw parameters and are calculated by counting pixels of rosette binary images and the edge pixels respectively and transformed to millimeters. **(B)** The parameters of roundness and roundness 2 and isotropy represented circular parameters. **(C)** Eccentricity and RMS were symmetric parameters. **(D)** Compactness and SOL were based on the center distance. Pink area around RMS, compactness and roundness 2 represent rosette convex hull, and for isotropy it represents rosette polygon. The characteristics of the parameters are described in detail in the Section Materials and Methods.

The plant phenotyping facility at the University of Helsinki Viikki campus (http://blogs.helsinki.fi/nappi-blog/) was used for the phenotypic characterization of the selected *Arabidopsis* mutant collection. The plants were imaged daily by overhead CCD camera for RGB images positioned in a PlantScreen™ analysis chamber with automated plant transportation between the imaging, weighing and watering stations. The RGB images were obtained for 20 plants at the time and stored in central database. The images were pre-processed online as described in Awlia et al. (2016) to allow collecting binary and RGB data for each plant. The obtained binary images were used for calculating growth parameters of area and perimeter. The obtained parameters of area, perimeter and the convex hull were then used for automatic online calculations of morphometric rosette parameters including: roundness1, roundness2, isotropy, eccentricity, compactness, Rotational Mass Symmetry (RMS), and Slenderness of Leaves (SOL) (PlantScreen™ analyzer, PSI, Czech R.). To characterize the general morphology of the mutant lines these nine morphological parameters were grouped into four categories based on their type: raw, circularity, symmetry and center distance, and compared over time (Figure 1). Raw parameters were represented by area and perimeter of the rosette and they were calculated by counting pixels of a rosette picture and the edge pixels respectively and transformed to millimeters (Figure 1A). The parameters of roundness1 and roundness2 and isotropy represented the circular parameters (Figure 1B). The parameter roundness describes rosette area in comparison to perfect circle with same perimeter and is affected by leaf slenderness, petiole length and leaf perimeter. For wild type plant, this parameter usually takes values between 0.1 and 0.5 while a perfect circle has value 1. Roundness value tends to decay overtime due to leaf development that at the same time increases the rosette perimeter. Roundness 2 uses rosette convex hull area and perimeter for its computation and for wild type plants this parameter appears to have values between 0.7 and 1.0 following an oscillating pattern with less steep peaks over time (Figure 1B). Isotropy uses the area of a drawn polygon on top of the rosette (Figure 1B). Thus, isotropy has a behavior similar to roundness 2 over time, but with less steep peaks and decreasing tendency similar to roundness. The eccentricity and RMS were symmetric parameters (Figure 1C). Eccentricity describes how elliptical the plant rosette is, where a value close to 1 corresponds to a rosette with highly sharp elliptical shape, while a value close to 0 describes a circular shape. Wild type rosette shows a high eccentricity peak that decays over time with a second smaller peak by the end of growth, thus the rosette shape fluctuates between a round and an elliptical shape. On the other hand, RMS describes the symmetry of the plant rosette by making a ratio between the non-overlapping rosette convex hull area and a perfect circle of the same area centered in the plant centroid and the overlapping area of both. RMS shows a similar pattern as eccentricity, but with higher absolute values and a sharper peak. Compactness and SOL were based on the center distance (Figure 1D). Compactness is the ratio between the rosette area and the rosette convex hull area. This parameter tells about petiole length and leaf blade width. The parameter SOL explains how sharp the leaf blades are, but it is also affected by the leaf number. SOL was derived from the ratio between squared rosette skeleton and rosette area. Thus, SOL can take values greater than 0 and below 50 in dimensionless units for wild type plants (Figure 1D).

### Experimental design

Ten days old (10 DAS) *Arabidopsis* plants were subjected to growth and morphological characterization by top view imaging for the following 10 days. One phenotyping round was designed to accommodate a maximum of 960 *Arabidopsis* plants representing 36 genotypes at a time in six consecutive experimental rounds called F006–F011. The total number of lines analyzed throughout the six rounds was 43. The maximum of 36 genotypes were divided in three batches that were rotating between the growth area and the PlantScreen™ analysis chamber. Each batch consisted of three experimental units of four mutant genotypes randomized with Col-0, each represented by 20 individual plants. One experiment consisted thus of five trays of altogether 100 plants. Each experimental unit had their own Col-0 wild type in randomized block design to normalize for any local differences in the microenvironments of the PlantScreen™ or the growth area. Each line showing any phenotypic responses was analyzed in at least three independent experimental rounds. Lines that did not show differences as compared to the Col-0 wild type were excluded from the subsequent rounds thus resulting in reduced numbers of genotypes included.

### Phenotypic analysis of flowering time and flower structures

After the image based growth and morphological measurements of the 20 mutant and 20 Col-0 plants in the PlantScreen™ system, the flowering time parameters leaf numbers at bolting (LAB) and days to bolting (DTB), were manually counted for each of the plant individuals. The number of rosette leaves were counted at the appearance of the flower bud (developmental stage 5.10, Boyes et al., 2001) and the DTB was recorded at the same time. The flowering time phenotypes were observed in two to three independent experimental rounds. Finally, flowers of the main inflorescences were photographed and further dissected for floral organ analysis under stereomicroscope (SteREO Discovery.V20, Zeiss). Microscopic pictures of the inflorescence tips, single flowers, sepals and petals were taken with the attached camera (AxioCam ICc3, Zeiss). The analyzed inflorescences and flowers originated from several independent experiments. Flower developmental stages were determined as in Smyth et al. (1990). To confirm pollen viability pollen grain staining according to the modified Alexander method was performed (Peterson et al., 2010). Anther images were captured using Leica DFC420 C camera attached to an optical microscope.

### Statistical analysis

The significance of the differences between mutant lines and Col-0 was computed by contrasting two fitted models to the data points using several order polynomials (Mirman, 2014). First, a model was fitted to all data points and then a second model was fitted including the factor genotype (wild type and mutant). These two models were then compared using the Chi square test to determine if the second model explained more variance than the first one beyond the significance (α = 0.05). If the second model was statistically different from the first one, it implied that the compared genotypes were different. These statistical analyses were conducted in R software (https://www.r-project.org/). LAB and DTB analysis were performed by Analysis of Variance using GLM procedure and pairwise comparisons against Col-0 using option Dunnett in the MEANS statement using SAS/STAT© software version 9.4 (SAS Institute Inc., Cary, NC, USA).

## Results

### Curation of the *Arabidopsis* RING-type ubiquitin E3 ligase protein sequences

**Figure 2:**
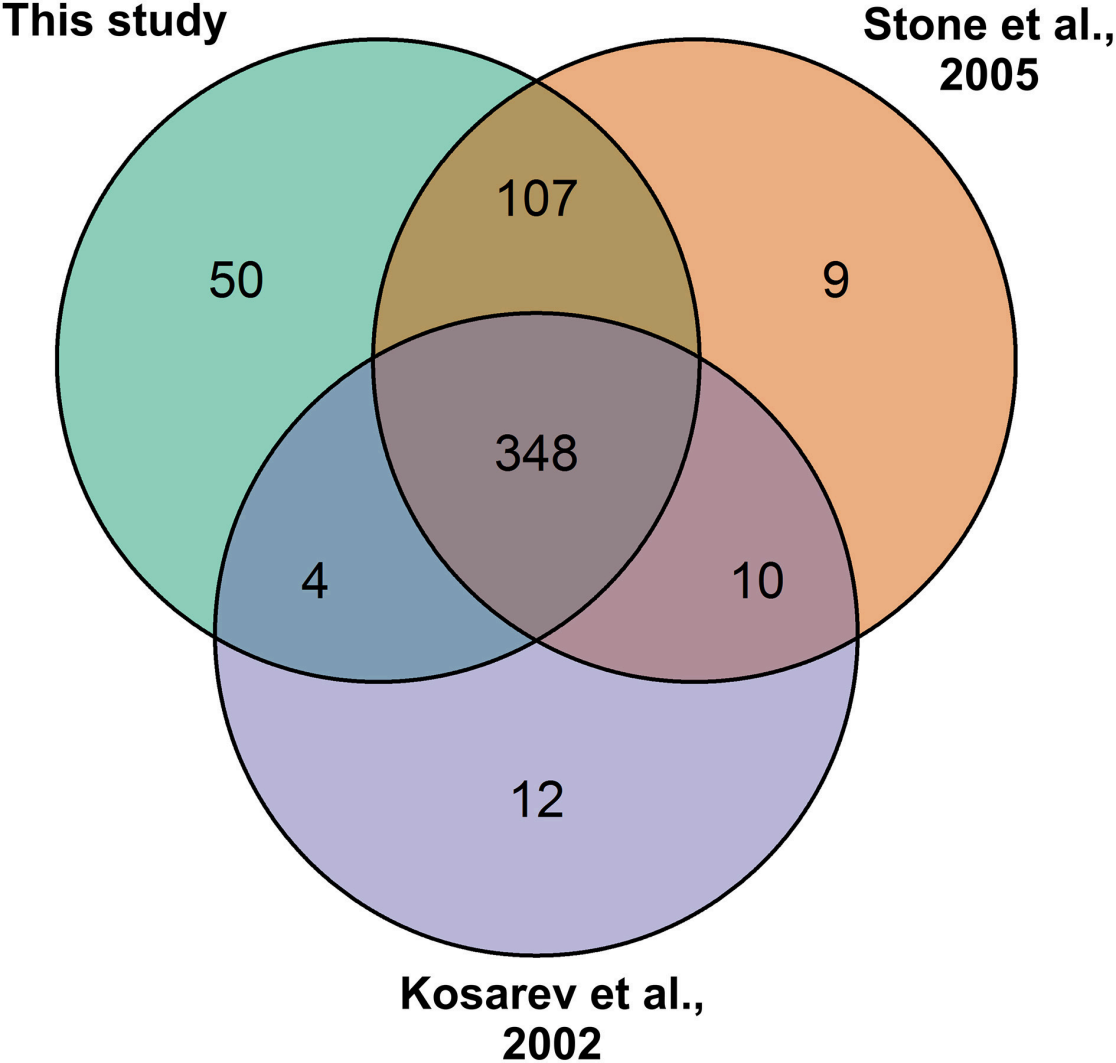
**RING gene family of 509 as identified by: this study (Kosarev et al., 2002; Stone et al., 2005)**. This study brought in 50 new RING genes and 31 from the earlier studies were excluded.

To re-confirm the published RING-type ubiquitin E3 ligase proteins encoded in *Arabidopsis* genome the 27,667 *Arabidopsis* proteins from the latest genome annotation release (ARA11) were scanned for RING domains. Through filtering the signatures in Gene3D, SUPERFAMILY, ProSiteProfiles, SMART, Pfam, ProSitePatterns altogether 509 putative RING domain containing protein sequences were obtained (Supplemental Table 2). RING gene names and descriptions were obtained from Araport using Thalemine tool (Supplemental Table 2). Araport used curated but also automatic gene annotation, therefore many RING domain containing proteins were annotated as RING/U-box protein although they did not contain U-box domain. Similarly, some were annotated as RING/FYVE/PHD zinc finger superfamily proteins. The 509 RING sequences were compared to the previously described RING-type protein sequences (Kosarev et al., 2002; Stone et al., 2005). From the 509 identified RING domain proteins 457 matched with the 490 previously described, thus resulting in 31 non-matching sequences (Figure 2). These non-matching sequences were thoroughly analyzed and, 6 of them were found to be merged with other gene models, 10 had no RING domain, 3 were not found in the database, 3 corresponded to pseudogenes, 7 were split and a new locus identifier had been assigned for them, and 2 were transposable elements (Supplemental Table 3A). The 50 additionally identified RING domain proteins were shown to represent diverse RING domains such as, 1 of D type, 4 of C2 type, 20 of H2, 16 of HC, 2 of S/T, and 7 of V (CH) type, according to the Stone et al. (2005) classification (Supplemental Table 2).

### Differential gene expression data identifies 122 flower related RING ubiquitin E3 ligases

**Figure 3:**
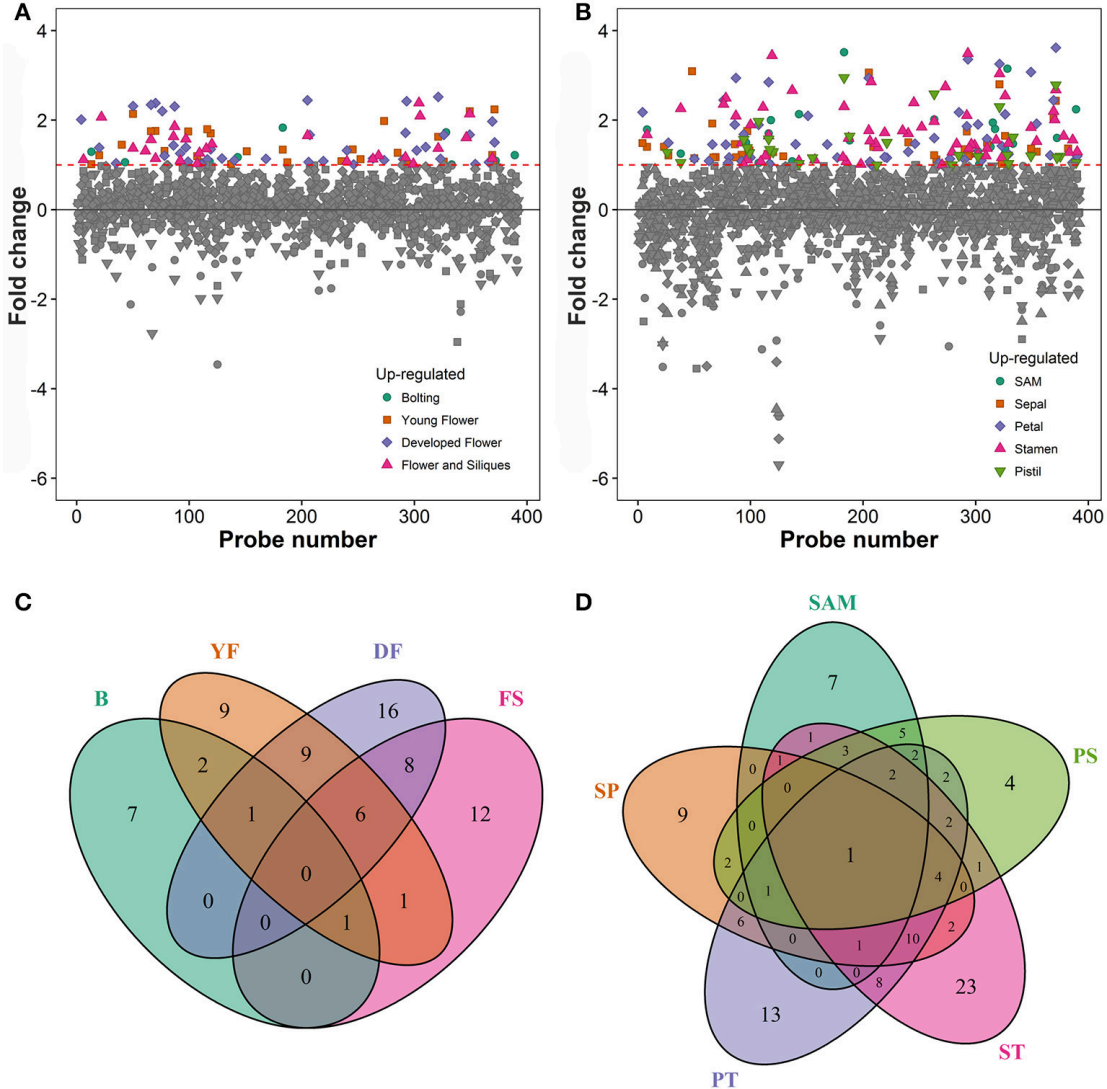
**(A)** Differential expression profiles of developmental stage enriched RING genes relative to developed rosette. **(B)** Differential expression profiles of flower organ enriched RING genes relative to rosette; SAM, Shoot apical meristem. **(C)** Venn diagram of RING genes expressed in the different developmental stages (B, bolting; YF, Young flower; DF, Developed flower; FS, Flowers and siliques). **(D)** Venn diagram of RING genes expressed in the different flower organs; SAM, Shoot apical meristem; SP, Sepal; PT, Petal; ST, stamen; PS, pistil.

To associate the RING domain proteins with flowering or flower development two approaches were followed: (1) identifying those with gene expression enhanced or enriched during flower development or in flower organs, and (2) by searching RING proteins interacting with known flower regulators. For the first approach Genevestigator (Hruz et al., 2008) tool was used to rank differentially expressed genes (DEG) of the identified RING genes over *Arabidopsis* developmental stages and in flower organs relative to their expression in developed rosette (Figures 3A,B). In the selected experiments in Genevestigator database, probes were available for 393 of the 509 RING E3 ligases analyzed. The cut off for DEGs was set at 2-fold to be included in the selection resulting in lists of genes of interest for each of the categories. This process was repeated to identify gene expression enrichment at each of the development stages of bolting, young flower, developed flower, and flower and siliques. For the developmental categories altogether 71 DEGs were identified (Figure 3C). In addition to the developmental stages, enrichment for shoot apical meristem, sepal, petal, stamen and pistil organs were retrieved and resulted in 109 DEGs (Figure 3D). Some of the RING genes were common between these two categories and in total 122 unique RING genes were up-regulated in the flower related processes. The gene identifying AGI codes of these 122 flower related candidates are provided in the Supplemental Table 3B.

For the second approach we identified 6 additional genes of interest through literature study and from interaction networks of CO, COP1, and TOE2 from BioGRID (http://thebiogrid.org/). Based on these interaction screens 5 RING E3 ligases were selected to the study, represented by the following mutant lines; N656705 (AT5G65683), N686069 (AT1G61620), N372291 (AT3G29270), N2037522 and N67002 (AT4G17680), and N742646 (AT2G44410). In addition, a mutant line for COP1, *cop1-6*, and RED AND FAR-RED INSENSITIVE 2 (RFI2) for which a role in mediating red and far-red light signaling and ubiquitination activity has been shown *in vitro*, were included (Stone et al., 2005; Chen and Ni, 2006a). This E3 ligase was selected as a candidate since its expression is regulated by circadian clock and *rfi2-1* mutant flowers early (Chen and Ni, 2006b). Thus, one mutant allele for RFI2 (N878610) was included in the study. Mutants representing these genes were analyzed together with the flower up-regulated RINGs and were named flower related UPS candidates in the Supplemental Table 1.

### Representative mutant collection

For functional characterization of the 122 flower related UPS candidates and those selected based on literature, a mutant collection was obtained from the NASC stock center. The mutants represented lines from CATMA, SAIL, SALK, and GABI-Kat collections (Alonso et al., 2003; Rosso et al., 2003; Schmid et al., 2005; Kleinboelting et al., 2012). Altogether 43 lines were shown to contain T-DNA insertion in one locus, six were doubtful and were omitted from the analysis. To confirm that the T-DNA insertion had interrupted the gene of interest, their altered expression levels were confirmed by qPCR analysis with primers listed in Supplemental Table 1. For 43 accessions representing 30 unique loci from the 122 flower related UPS candidates and the selected candidates a differential gene expression pattern was analyzed. Altogether 19 lines were knock-outs, and 13 knock-down mutants, and for 10 lines up-regulation of the gene of interested was observed (Supplemental Table 1). For one line, no differential expression was confirmed and this was excluded from the phenotyping. For 14 lines alleles were available with similar or opposite gene expression patterns.

### Phenotypic screen of the mutant accessions

**Figure 4:**
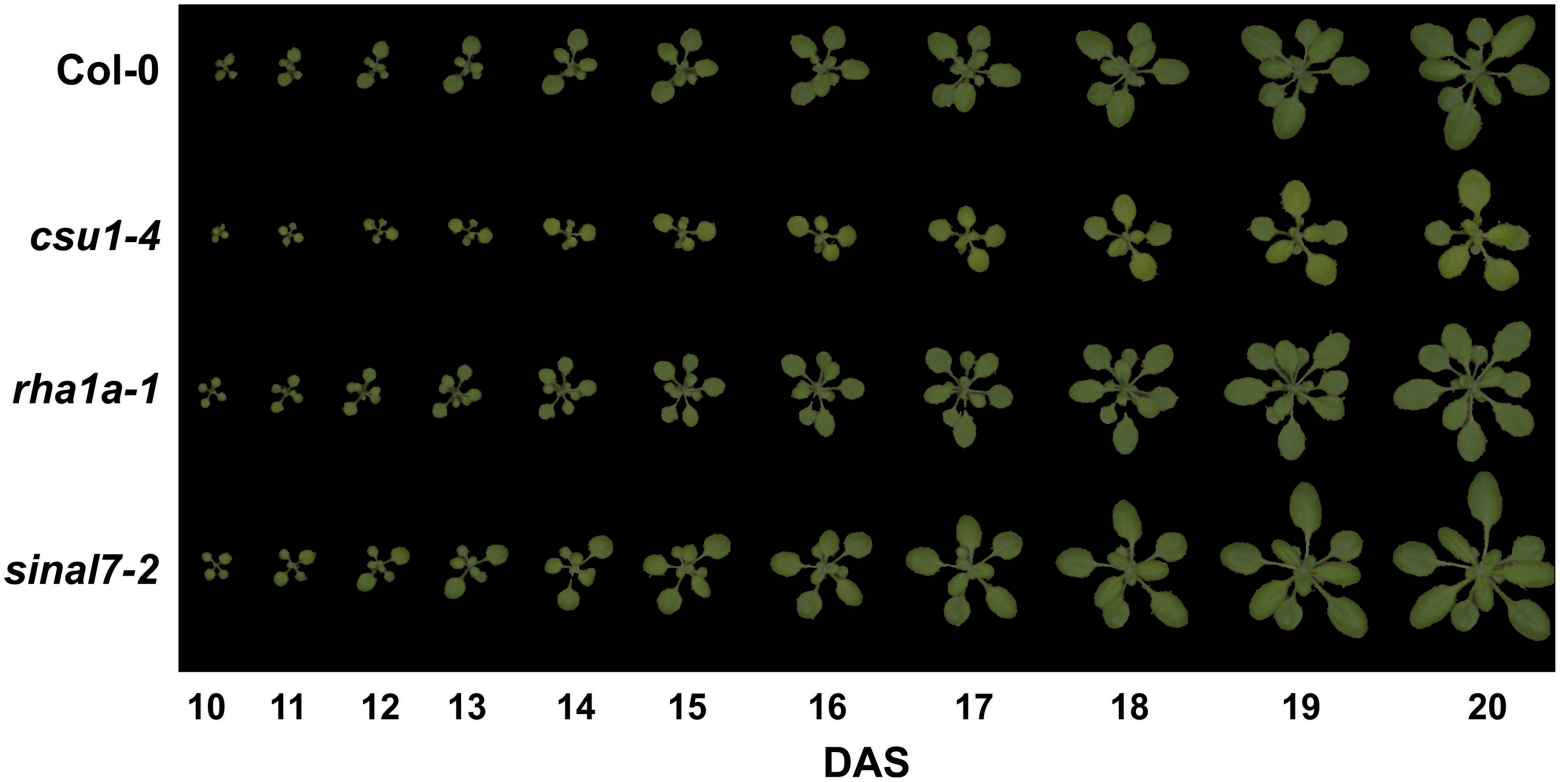
**Rosette growth of *csu1-4, rha1a-1* and *sinal7-2* mutants**. Representative rosette images are shown from days 10 to 20 after stratification. DAS, days after stratification.

From the genotypically and qPCR confirmed T-DNA insertion mutant lines, 43 were subjected to phenotypic characterization by top view RGB imaging using the PlantScreen™ system. Image series of each analyzed line were collected daily allowing analyzing the growth and changes in morphology over time. For scoring those lines showing phenotypes, we fitted general additive models (GAM) to each parameter of each analyzed lines (data not shown). Most of the lines showed no differences to their corresponding Col-0 controls. However, three lines were consistently different across the experiments compared to Col-0 in both growth and rosette morphology: *csu1-4* (*cop1 suppressor 1-4*, N686069), *sinal7-2* (*seven in absentia like 7-2*, N833574) (Peralta et al., 2016) and *rha1a-1* (*ring-h2 finger a1a-1*, N2045046) (Table 1, Supplemental Tables 1, 4). The *csu1-4* mutant rosette was clearly smaller than Col-0 and showed a yellowish coloration (Figure 4). The mutant line *rha1a-1* seemed to have smaller leaves than Col-0, however, at the end of growth it appeared to have more leaves that resulted in similar final rosette area as compared to Col-0. This line also had shorter petioles and leaf serration. The third line *sinal7-2* rosette was clearly larger than Col-0 but did not show major differences in color, shape or number of leaves (Figure 4).

**Table 1:** **Polynomial order and their respective Chi square probability from ANOVA test for each parameter used in this study**.

**Parameter**	**Polynomial order used**	Chi square probability^*^		
		***csu1-4***	***rha1a-1***	***sinal7-2***
Area	3	<2.2e-16^***^	<2.2e-16^***^	<2.2e-16^***^
Perimeter	3	9.116e-15^***^	9.076e-11^***^	1.506e-10^***^
Compactness	4	<2.2e-16^***^	1.274e-10^***^	1.132e-06^***^
Roundness	5	<2.2e-16^***^	2.665e-15^***^	1.613e-06^***^
Roundness 2	5	<2.2e-16^***^	<2.2e-16^***^	8.43e-06^***^
Isotropy	6	0.0008271^***^	8.16e-08^***^	5.058e-05^***^
Eccentricity	6	<2.2e-16^***^	<2.2e-16^***^	5.102e-14^***^
RMS	6	<2.2e-16^***^	<2.2e-16^***^	4.53e-05^***^
SOL	3	0.0006792^***^	3.867e-07^***^	0.006651^**^

^*^Comparison was performed using an ANOVA test between a base model and a model including the genetic background as factor.

*Significance codes*:

*** *p < 0.001*;

** *p < 0.01*;

* *p < 0.05*.

*Base model = Parameter ~ polynomial of Day + Random factor Day and Plant ID*.

Model = Parameter ~ polynomial of Day ^*^ genetic background (Col-0 or knockout line) + Random factor Day and Plant ID.

**Figure 5:**
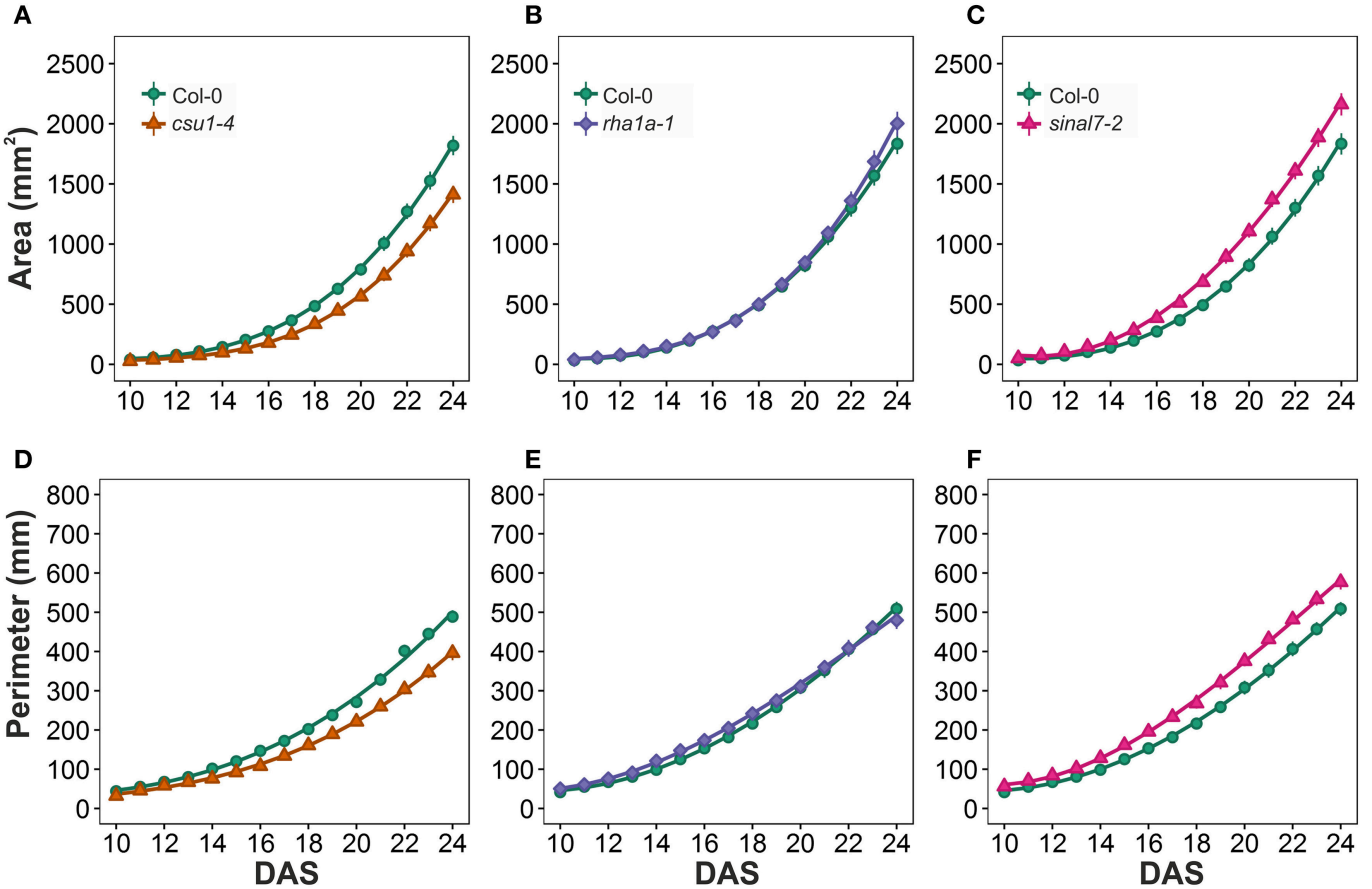
**Growth measurements from day 10 to 20**. Area **(A)** *csu1-4*, **(B)** *rha1a-1*, and **(C)** *sinal7-2*. Perimeter **(D)** *csu1-4*, **(E)** *rha1a-1*, and **(F)** *sinal7-2*. Markers, daily mean; error bars, 95% confidence interval; Curves, fitted models; *n* = 20 plants. The experiment was repeated at least 3 times with similar results. DAS, days after stratification.

To further analyze these three lines, mixed non-linear models were fitted to their data using several order polynomials for parametric analysis of the models. This analysis confirmed the earlier observations of significant changes in growth and development for these lines over time (Table 1). Line *csu1-4* showed slower growth, reduced rosette area and perimeter compared to Col-0 along the complete measured period (Figures 5A,D). For line *rha1a-1* the rosette area was very similar to Col-0 being, however, slightly but significantly larger over time probably due to its higher number of leaves (Figure 5B, Table 1). Although the differences between *rha1a-1* and Col-0 were small the statistical model was able to capture those. Conversely, *sinal7-2* showed both area and perimeter larger than Col-0 indicating more vigorous growth (Figures 5C,F).

### Circularity parameters

**Figure 6:**
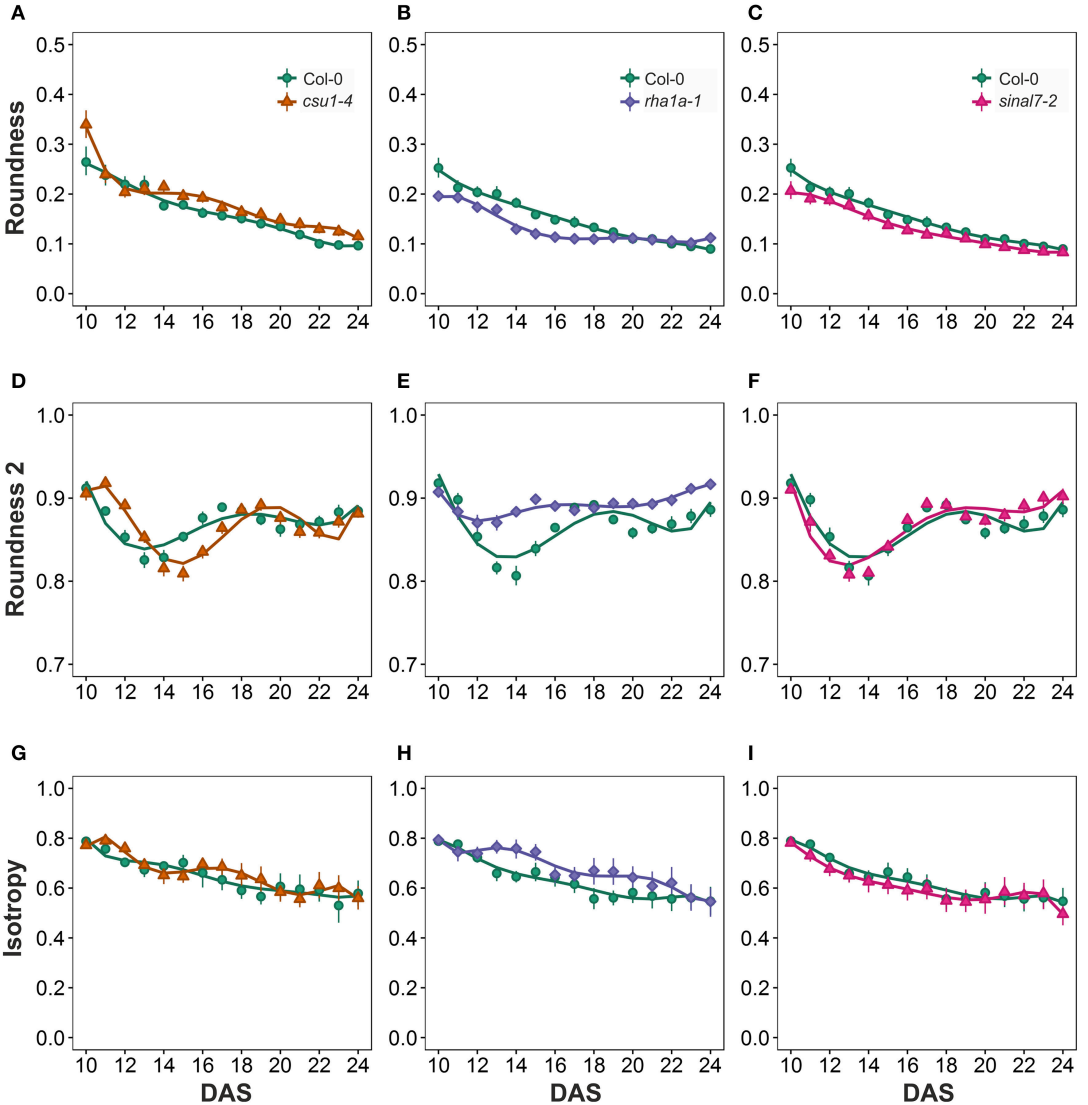
**Circularity measurements from day 10 to 20**. Roundness **(A)** *csu1-4*, **(B)** *rha1a-1*, and **(C)** *sinal7-2*. Roundness2 **(D)** *csu1-4*, **(E)** *rha1a-1*, and **(F)** *sinal7-2*. Isotropy **(G)** *csu1-4*, **(H)** *rha1a-1*, and **(I)** *sinal7-2*. Markers, daily mean; error bars, 95% confidence interval; Curves = fitted models; *n* = 20 plants. The experiment was repeated at least 3 times with similar results. DAS, days after stratification.

Morphological data for parameters of circularity that include roundness, roundness 2 and isotropy were also evaluated for these lines (Figure 1). Line *csu1-4* showed increased roundness over the total period analyzed in comparison to Col-0 (Figures 6A–C, Table 1). However, *csu1-4* roundness curve had similar pattern to Col-0 but shifted to the right (Figure 6A). Similar situation was observed for *sinal7-2*, where the roundness curve shape was almost identical to Col-0 but in this case was shifted to the left, showing lower roundness along the total time period (Figure 6C, Table 1). Roundness curve of *rha1a-1* was neither shifted nor similar to Col-0 curve. This line showed a lower roundness than Col-0 at the beginning of the analysis, reaching a stabilization point around 16 DAS (Table 1). For Col-0 plants roundness continued decreasing until it become lower than *rha1a-1* (Figure 6B).

Line *csu1-4* showed a similar roundness 2 pattern as Col-0 that is shifted to the right by approximately 2 days (Figures 6D–F). Line *rha1a-1*, showed an oscillating pattern too, however, its roundness 2 values were constantly close to 0.9 with less steep peaks than Col-0, presenting the highest differences between days 12 and 16 (Figure 6E, Table 1). Similarly, to line *csu1-4*, line *sinal7-2* presented an oscillating pattern very similar to Col-0, however, this time the curve had shifted to the left by approximately 1 day (Figure 6F).

Isotropy showed similar results as roundness and roundness 2, where line *csu1-4* and *sinal7-2* had similar oscillating pattern as Col-0, but *csu1-4* curve is shifted to the right, while the curve for *sinal7-2* is shifted to the left (Figures 6G–I). Line *rha1a-1* showed a constant high isotropy value decreasing over time until reaching Col-0 pattern by day 23 (Figure 6H, Table 1).

### Symmetry parameters

**Figure 7:**
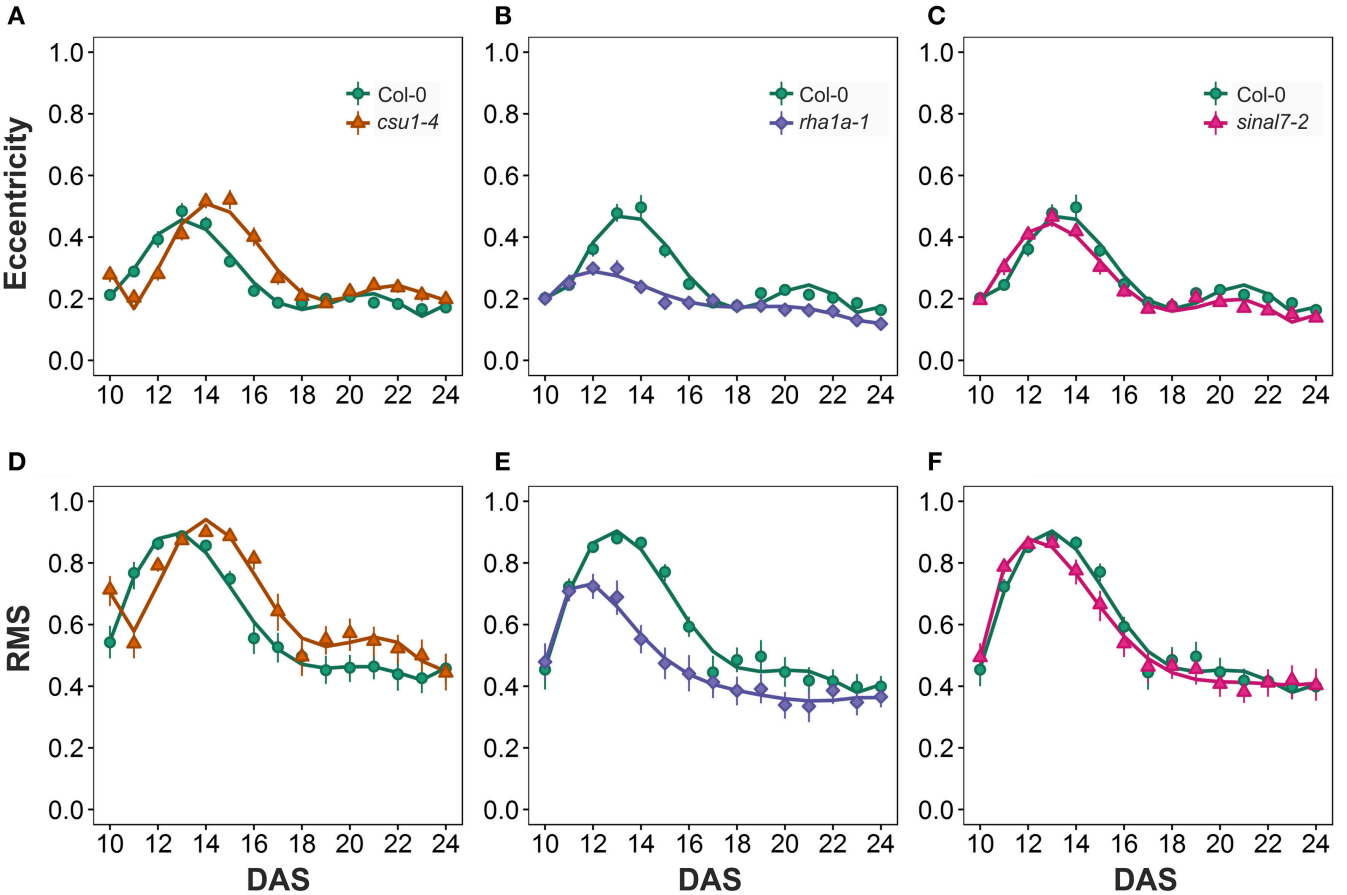
**Symmetry measurements from day 10 to 20**. Eccentricity **(A)** *csu1-4*, **(B)** *rha1a-1*, and **(C)** *sinal7-2*. Rotational Mass Symmetry (RMS) **(D)** *csu1-4*, **(E)** *rha1a-1*, and **(F)** *sinal7-2*. Markers, daily mean; error bars, 95% confidence interval; Curves, fitted models; *n* = 20 plants. The experiment was repeated at least 3 times with similar results. DAS, days after stratification.

The morphological parameters describing symmetry were eccentricity and rotational mass symmetry (RMS) (Figure 1). For eccentricity, line *csu1-4* showed a similar pattern as Col-0 plants with a large and a small eccentricity peak, but shifted to the right (Figure 7A). Line *rha1a-1* presented no shift in its curve, but it showed a rather flat peak around days 11 and 15, remaining lower than Col-0 until the end of the analysis (Figure 7B, Table 1). This result shows that *rha1a-1* is less eccentric than Col-0 along the complete analysis. Line *sinal7-2* showed also a similar pattern to Col-0 plants with two eccentric peaks, but slightly shifted to the left (Figure 7C). For RMS line *csu1-4* showed similar pattern as Col-0 plants, but shifted again to the right about 1 day for the highest peak and remained higher than Col-0 in the last days of the analysis (Figure 7D). On the other hand, *rha1a-1* presented no shift in its curve, but it showed a decrease in the peak around days 11 and 15, decaying faster and remaining lower than Col-0 plants (Figure 7E, Table 1). Like in eccentricity, *sinal7-2* was almost indistinguishable from the Col-0 plants, except for a slight shift to the left captured by the model (Figure 7F).

### Center distance parameters

**Figure 8:**
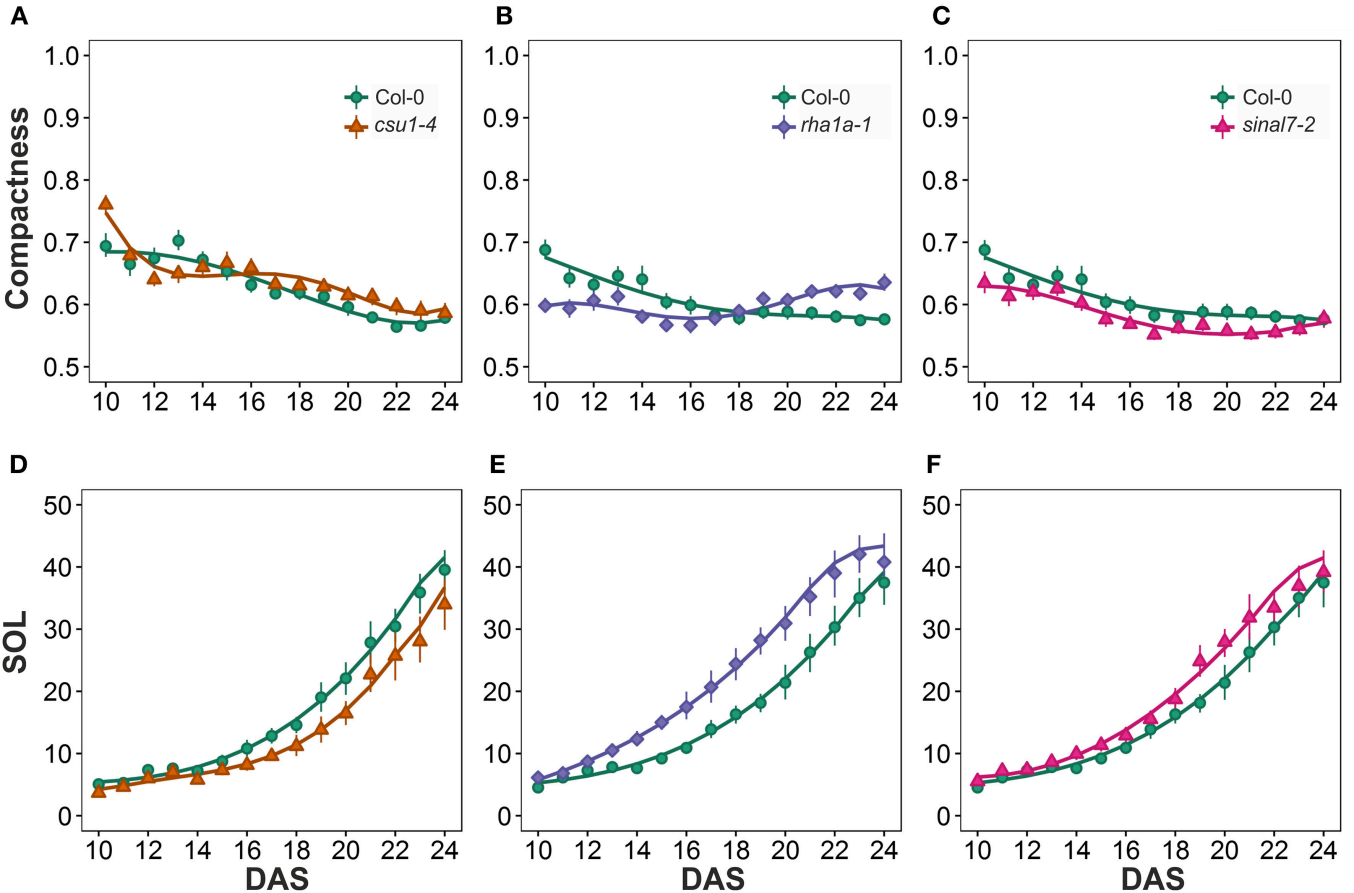
**Center distance measurements from day 10 to 20**. Compactness **(A)** *csu1-4*, **(B)** *rha1a-1*, and **(C)** *sinal7-2*. Slenderness Of Leaves (SOL) **(D)** *csu1-4*, **(E)** *rha1a-1*, and **(F)** *sinal7-2*. Markers, daily mean; error bars, 95% confidence interval; Curves, fitted models; *n* = 20 plants. The experiment was repeated at least 3 times with similar results. DAS, days after stratification.

The last two morphological parameters analyzed were compactness and slenderness of the leaves (SOL), which were based on the center distance (Figure 1). Here line *csu1-4* showed a decay of compactness overtime in a similar way to Col-0 plants, but its curve was shifted to the right (Figure 8A). Lines *csu1-4* and *sinal7-2* presented quite normal compactness curves, while for *rha1a-1* the pattern that was less compact than Col-0 plants at the beginning of the analyzed period (Figure 8B, Table 1). The compactness later rises above Col-0, showing higher compactness values. Like for the previously described parameters, *sinal7-2* compactness curve showed slightly lower values than Col-0, except for the last 2 days where Col-0 plants reached *sinal7-2* compactness (Figure 8C).

Line *csu1-4* showed lower SOL values than Col-0, while *rha1a-1* and *sinal7-2* showed higher SOL values than Col-0 (Figures 8E,F, Table 1). The main differences in SOL could be observed during the exponential growing phase of the rosette and reaching a plateau at the end of the analyzed period where the differences to Col-0 plants become insignificant (Figures 8D–F).

### Flowering time phenotypes

Flowering time mutants identified in the screen represented both with reduced and increased leaf numbers at bolting (Table 2). Line *csu1-4* (AT1G61620) was clearly early-flowering in both experimental replications. AT5G63970, a putative forkhead box protein, mutant line was early flowering in one of two experimental replications. SBP (S-ribonuclease binding protein) family protein (AT4G17680) was late flowering in both experimental replications. As already shown by others, *cop1-6* mutant was early flowering in both LAB (7) and DTB (22). In most of the mutant lines, LAB did not differ from Col-0 in all experimental replications, but the trend was observed in both or all. LAB or DTB of *sinal7-2* did not differ from Col-0 in either of the experimental replications.

**Table 2:** **Number of leaves and number of days to bolting in *Arabidopsis* mutant lines grown in LDs**.

**AGI**	**NASC ID**	**Leaves at bolting**			**Days to bolting**		
		**Mutant line**	**Dunnett**	**Col-0**	**Mutant line**	**Dunnett**	**Col-0**
AT1G61620	N686069	9.7 ± 2.1	^*^	13.5 ± 1.8	25.2 ± 1.6		25.7 ± 2.2
*CSU1*	*csu1-4*	9.9 ± 1.5	^*^	15.5 ± 1.1	23.6 ± 1.6	^*^	26.8 ± 0.7
		12.1 ± 0.4	^*^	15.1 ± 0.4	24.9 ± 0.6	^*^	26.2 ± 0.7
AT1G68820	N667194	15.8 ± 1.5	^*^	13.5 ± 2.0	27.2 ± 1.5	^*^	25.3 ± 1.6
*RING E3*		16.0 ± 1.2		16.6 ± 1.4	28.3 ± 1.2		28.0 ± 1.1
		14.3 ± 0.7	^*^	15.9 ± 0.3	24.9 ± 0.9	^*^	27.2 ± 0.6
	N659628	14.1 ± 1.8		13.6 ± 2.0	26.9 ± 1.6	^*^	25.5 ± 1.7
		16.5 ± 1.1		15.4 ± 0.9	28.2 ± 1.1	^*^	26.7 ± 1.3
		16.1 ± 1.3		16.6 ± 1.4	27.9 ± 1.1		28.0 ± 1.1
AT2G22680	N219963	16.4 ± 1.0	^*^	12.6 ± 1.7	27.9 ± 1.3	^*^	25.2 ± 1.4
*WAVH1*		16.6 ± 1.3		15.6 ± 1.7	27.7 ± 1.2	^*^	26.1 ± 1.2
	N653622	13.3 ± 2.0		12.5 ± 1.9	25.0 ± 1.5		25.0 ± 1.6
		15.5 ± 1.0		14.6 ± 1.4	26.2 ± 1.0		25.4 ± 1.5
AT2G32950		7.5 ± 0.6	^*^	12.6 ± 0.7	22.7 ± 1.4	^*^	28.4 ± 0.9
*COP1*	*cop1-6*	6.7 ± 0.7	^*^	13.7 ± 0.3	21.1 ± 0.6	^*^	27.3 ± 0.9
AT2G37150	N685421	15.3 ± 1.5	^*^	13.5 ± 2.0	27.2 ± 1.1	^*^	25.3 ± 1.6
*RING E3*		16.4 ± 1.9		15.4 ± 0.9	27.0 ± 1.3		26.7 ± 1.3
		16.6 ± 1.3		16.6 ± 1.4	28.3 ± 0.8		28.0 ± 1.1
AT2G47700	N878610	15.5 ± 2.1	^*^	13.0 ± 1.4	26.6 ± 1.5		25.6 ± 1.3
*RFI2*	*rfi2-3*	16.5 ± 1.4		15.5 ± 1.1	27.7 ± 0.9		26.8 ± 0.7
AT3G07200	N481270	15.3 ± 2.0	^*^	13.5 ± 1.8	27.7 ± 1.7	^*^	25.7 ± 2.2
*STUBL3*	*stubl3*	15.6 ± 2.0		15.5 ± 1.1	27.3 ± 1.7		26.8 ± 0.7
AT3G09760	N653280	13.0 ± 1.8		12.6 ± 1.7	24.8 ± 1.4		25.2 ± 1.4
*RING E3*		13.9 ± 1.5		14.6 ± 1.4	23.9 ± 1.3	^*^	25.4 ± 1.5
AT4G17680	N67002	15.2 ± 2.1	^*^	13.5 ± 1.8	27.2 ± 1.7	^*^	25.7 ± 2.2
*SBP*		16.8 ± 1.3	^*^	15.5 ± 1.1	27.9 ± 1.3	^*^	26.8 ± 0.7
AT5G37890	N596989	13.1 ± 2.1		13.0 ± 1.4	25.6 ± 1.9		25.6 ± 1.3
*SINAL7*	*sinal7-2*	14.4 ± 1.7		15.1 ± 1.3	25.7 ± 1.3		26.6 ± 1.4
	N833574	14.0 ± 0.7	^*^	15.1 ± 0.4	24.3 ± 0.5	^*^	26.2 ± 0.7
AT5G63970	N694155	13.3 ± 2.2		13.6 ± 2.0	25.6 ± 1.8		25.5 ± 1.7
*RING E3*		13.5 ± 1.3	^*^	15.4 ± 0.9	24.6 ± 1.5	^*^	26.7 ± 1.3
		14.4 ± 0.7		15.1 ± 0.4	24.8 ± 1.0	^*^	26.2 ± 0.7

*Pairwise comparisons were performed against corresponding Col-0-line using Dunnett's test*.

* *Indicates statistically significant difference (α = 0.05)*.

*N = 19–20 in each row*.

### Mutation in *SINAL7* causes flower growth phenotypes

**Figure 9:**
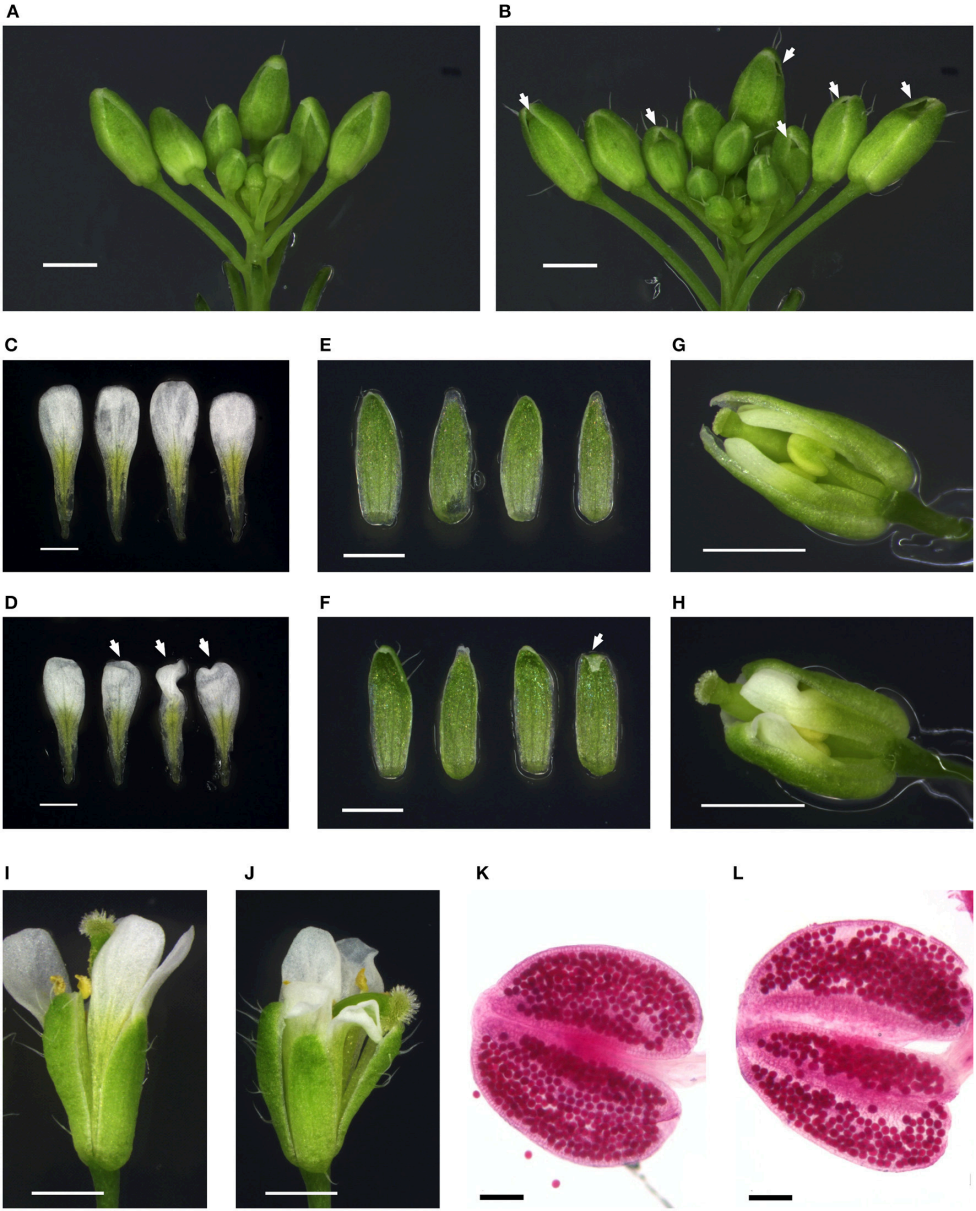
**Flower phenotypes of the *sinal7-2* mutant**. Flower developmental stages assigned according to Smyth et al. (1990). Scale bars: 1 mm **(A–J)** and 100 μm **(K,L)**. **(A,B)** Representative inflorescences of Col-0 **(A)** and *sinal7-2* **(B)**. All flowers and siliques older than stage 12 have been removed. Mutant flower buds contain cavities beneath the bud tip (indicated with white arrows). **(C,D)** Petals of a Col-0 **(C)** and a *sinal7-2* **(D)** flower at stage 15. White arrows pointing at the wrinkled mutant petals. **(E,F)** Adaxial surface of the sepals from a Col-0 **(E)** and a *sinal7-2* **(F)** flower at stage 15. White arrow pointing at the bending lateral sepal tip of *sinal7-2*. **(G,H)** Late stage 12 flower buds of Col-0 **(G)** and *sinal7-2* **(H)**. The medial sepals have been removed to reveal the elongating and wrinkling petals blocked by the ingrown lateral sepals of the mutant. **(I,J)** Col-0 **(I)** and *sinal7-2* **(J)** flowers stage 15. **(K,L)** Representative anthers from Col-0 **(K)** and *sinal7-2* **(L)** flowers stage 12–13 stained for pollen viability.

Flower morphology of the analyzed mutants was observed under stereomicroscope. The mutant line *sinal7-2* was found to produce flower buds of abnormal shape, characterized by presence of cavities in the bud tips (Figures 9A,B). These openings were present at one or both sides of the affected buds and were caused by tips of the lateral sepals bending inwards (Figures 9E,F). Also medial sepals frequently showed altered morphology: their tips covered the buds to a lesser extent than in Columbia, resulting in their “blunt” appearance. Whereas these phenotypes were present in all 18 analyzed inflorescences of *sinal7-2* plants, regardless of the plant age—only two out of 13 analyzed wild type inflorescences showed similar sepal features, restricted to the first six flowers on the main stems. Scoring flowers stage late 12–15 (located between positions 1st and 20th on the main inflorescences) revealed that in 54% of the mutant flowers (43/80) at least one lateral sepal tip was bent inwards—as compared to 6/50, i.e., 12% in Col-0 (the analyzed flowers came from 13 to 9 individual plants, respectively).

Dissecting flower buds at the end of stage 12 revealed that the occurrence of ingrown lateral sepal tips was accompanied by petal wrinkling, as the sepal shape interfered with elongation of the petals (Figures 9G,H). Indeed, in some of the mature flowers with bent lateral sepal tips, the petal blades remained wrinkled; in several cases also pistil or stamen shape was affected (Figures 9C,D,I,J).

SINAL7 has been shown to mediate ubiquitination of glyceraldehyde-3-phosphate dehydrogenase 1 (GAPC1) enzyme *in vitro* and to affect its enzymatic activity and subcellular localization in *Arabidopsis* (Peralta et al., 2016). In plants lacking GAPC1 male sterility was observed (Rius et al., 2008). To investigate whether deficiency of SINAL7 impairs male fertility in the *sinal7-2* mutant, pollen viability was inspected according to the modified Alexander method (Peterson et al., 2010). Anthers of 12 mutant and 11 Col-0 flowers in the developmental stages late 12 and 13 were stained (early and late flowers, originating from at least five individual plants per line). However, no difference between the mutant and Col-0 pollen was observed: anthers of both lines contained almost exclusively viable pollen grains (Figures 9K,L).

## Discussion

Genomic knowledge in both model plants and crops is expanding at a fast pace. However, translating the knowledge from sequence to function and thereby from models to applications is hampered by bottlenecks in screening for the phenotypes associated with the genotypes. Here, we set out to conduct a reverse genetic approach (Bolle et al., 2011) by defining a proportion of the RING type ubiquitin E3 ligases to the developmental processes of flowering time control or flower development. To this end, the RING type ubiquitin E3 ligases were first curated in the most recent *Arabidopsis* genome annotation (ARA11) that had been improved, e.g., by the next generation sequencing techniques (Krishnakumar et al., 2015). Thereby, many gene models had indeed become obsolete, split, merged or their original sequence had changed. We also found that in the annotations there are a considerable number of RING domain containing proteins annotated as RING/U-box genes. RING and U-box share similar functions and are structurally and functionally similar, both are ubiquitin E3 ligases that work as scaffolds between the ubiquitin E2 conjugase and substrate. However, at the amino acid residual level RING and U-box domains are significantly different; in the RING domain the arrangement of cysteines and histidines mediate binding of two zinc ions to stabilize the RING domain, while the U-box domains are stabilized by a set of hydrogen bonds and salt bridges (Wiborg et al., 2008).

Recent studies have revealed complex molecular networks that include ubiquitin E3 ligases in regulation of flowering (Lazaro et al., 2012; Peng et al., 2013; Xia et al., 2013). To start defining the genomic flower related Ubiquitin Proteasome System of RING E3 ligases, we first verified the gene expression patterns of the curated RING genes. RING E3 ligases work at protein level but are likely to be transcriptionally directed to their relevant tissues. From the 509 RING genes, 122 were indeed associated with flowering with enrichment of gene expression in flower organs or during flowering. This observation prompted us to obtain a representative mutant collection for phenotypic evaluation.

To screen for phenotypes associated with the mutant collection, an automated plant phenotyping facility was utilized. To facilitate a phenotypic screening of a large *Arabidopsis* mutant collection a phenomics workflow was established to analyze simultaneously up to 36 genotypes in a PlantScreen™ imaging system installed at the Viikki campus of the University of Helsinki (http://blogs.helsinki.fi/nappi-blog/). Although T-DNA insertion knock-out mutants do not always impair gene function (Bolle et al., 2011), the high-throughput phenomics screen of altogether 43 genotypes singled out three mutant lines with clear growth and morphology phenotypes, three mutant lines with flowering time phenotypes and only one with altered flower structure.

For the *Arabidopsis* growth assessment, we analyzed rosette growth from 10 to 20 DAS. The analysis of such longitudinal data is challenging and demands automated statistical analysis and modeling steps. The rosette growth normally follows a sigmoid pattern showing a lag phase represented by slow growth around the first 10 days, accelerating in the middle and slowing down when getting close to the transition from the vegetative to reproductive phase. The best way to model data with sigmoid behavior is by fitting a three parameter logistic regression (3PL) to explain the three stages (Paine et al., 2012; Tessmer et al., 2013; Neilson et al., 2015). However, our analysis time window captured only the lag and the exponential phases, so a 3PL model was not suitable for our data. Therefore, we used polynomials for more flexibility and a better explanation of the data for all the parameters. This was particularly useful for the initial screening of the data of the tens of lines for the complex parameters like roundness, roundness 2, isotropy, compactness and RMS.

Typically, the parameters of roundness 2, isotropy and RMS increase and decrease over time. This behavior is due to the natural cycle of leaf initiation and expansion. At the beginning when the two first true leaves are developed, the rosette has an elliptical shape that becomes more circular when the leaves 3 and 4 appear and start to expand. Because leaves 3 and 4 keep on expanding, while the leaves 1 and 2 have already stopped expanding, the rosette takes an elliptical shape around day 12 (Figure 4). This process is repeated each time two new leaves develop and expand, explaining the oscillating behavior of these parameters. The steepness of each peak decrease over time because previously generated leaves expand making the rosette more circular. Thus, recording fluctuations in these parameters allows establishing the developmental timing of leaf initiation and expansion.

Here, three lines showed consistently significant differences in growth and morphology compared to the wild type Col-0. The mutant lines *csu1-4* and *sinal7-2* showed similar growth curve shapes as Col-0, but shifted to the left or right, respectively, for all morphological parameters. This behavior was explained by their speed of growth over time. If two lines differ in their growth rate but were analyzed only on one particular day after germination, they could show high differences in morphological parameters. Therefore, longitudinal time course analysis of *Arabidopsis* rosette growth and shape became compulsory for making accurate conclusions about the effect of a mutation also on morphology. On the contrary, the *rha1a-1* mutant did not show major differences in growth, but did for morphology. The increased number and serration of rosette leaves in *rha1a-1* rendered the rosette perimeter and the skeleton longer, thereby, reducing the roundness and increasing SOL during all time points (Figures 6B, 8E). Furthermore, the increased number of leaves of *rha1a-1* prevented its rosette from taking overly elliptical shape, keeping it more circular than Col-0 plants over time (Figure 4). This characteristic was translated in higher roundness 2, isotropy, compactness and lower eccentricity and RMS (Figures 6E–H, 7B–E, 8B). Thus, the morphological parameters can be used not only to record developmental timing but also to explain the plant architecture in a numeric manner.

The line showing an early flowering time phenotype was COP1 SUPPRESSOR1 (CSU1). *csu1-4* plants flowered three to six leaves earlier than Col-0 grown under LDs (Table 2). In addition to early flowering, *csu1-4* plants showed vegetative phenotypes: plants were smaller than Col-0 (Figure 5), the eccentricity, RMS and roundness2 development started later than Col-0 (Figures 6, 7), and SOL was smaller than in Col-0 (Figure 8). CSU1 has been shown to negatively regulate hypocotyls length in the dark, via ubiquitination of COP1 and repression of SPA1 (Xu et al., 2014). Our results indicate that CSU1 may regulate both vegetative and generative development. The line showing a late flowering phenotype, SBP family protein (AT4G17680), flowered one to two leaves later than Col-0 (Table 2). This gene was selected for the phenotypic analysis based on its interaction with TOE2. *toe2* is late flowering, and *toe1 toe2* double mutant represses *FT* expression (Zhai et al., 2015). Our results suggest that this SBP family protein could be involved in regulation of flowering time possibly through TOE2. Some SBP family members are known to regulate flowering time. Four SBP proteins, BOTRYTIS SUSCEPTIBLE1 INTERACTOR (BOI) and its three homologous repress flowering by repressing *FT* expression in a CO dependent manner and a CO independent manner via DELLA proteins (Nguyen et al., 2015). This evidence suggests that there might be a connection between SBP proteins and flowering time control.

In the *sinal7-2* mutant defects in flower morphology were observed. SINAL7 has been shown to ubiquitinate glyceraldehyde-3-phosphate dehydrogenase 1 (GAPC1) and to regulate its enzymatic activity and movement to nucleus (Peralta et al., 2016). GAPC1 plays a role in glycolysis, thus regulating carbon metabolism, and it has been also associated with cytoskeleton and mitochondria (Giegé et al., 2003; Anderson et al., 2004). *SINAL7* gene is differentially expressed in the *gapc1* knockout mutant as well as in *u-ATP9* plants with mitochondrial dysfunction (Rius et al., 2008; Busi et al., 2011). Although both *gapc1* and *u-ATP9* lines showed defects in male fertility (Gómez-Casati et al., 2002; Rius et al., 2008), we did not observe increased number of aborted pollen grains in the *sinal7-2* mutant (Figures 9K,L), suggesting that SINAL7-mediated GAPC1 regulation does not impact pollen maturation. Although we have not tested if the *sinal7-2* mutation influences pollen germination and pollen tube growth, fertility of the mutant did not seem to be strongly compromised. Instead, we observed defects in *sinal7-2* flower morphology - cavities in flower buds and wrinkled petals. Sepal curvature is controlled by giant cells in the abaxial epidermis, in which cell expansion is connected with endoreduplication (Roeder et al., 2010, 2012). A couple of mutants have been identified in which reduction of giant cells was accompanied by their sepals bending inwards. Closer examination of *sinal7-2* sepal epidermis will show whether the observed bent sepal tips and resulting flower bud cavities (Figures 9A,B,E–H) originate from endoreduplication defects, which could suggest a novel role for the SINAL7 protein. Other flower phenotypes of the mutant—wrinkling of petals as well as bending of stamens and pistils (Figures 9C,D,G–J)—seem to be a direct consequence of the abnormal shape of sepals posing an obstacle for the developing floral organs during their growth and release from the buds. Nevertheless, at this point it cannot be ruled out that the SINAL7 ubiquitin E3 ligase could be involved in the development of these flower organs in other ways.

Here we showed that automated, imaging based phenotyping platform is an efficient tool to overcome the limiting factors of manual and visual phenotypic measurements of large plant collections. Imaging based platforms also allow deep resolution of the phenotypes and thereby more precise association with the genotypes. Furthermore, the automated plant management and transportation to imaging, facilitates time course experiments. Thereby, recording longitudinal numeric values indicating changes in rosette size and morphology can be utilized in developmental timing of plant growth and development. Here the customized solution of the PSI PlantScreen™ system by top view CCD camera in combination with online data processing was used for high throughput phenotyping of an *Arabidopsis* mutant collection. The obtained resolution and high throughput, whereby hundreds of plants can be analyzed in the time that normally a handful would be analyzed, is an obvious advantage.

## Author contributions

MP conducted the genomic screens, performed the phenomic screens and statistical analysis of these data. KM performed the QPCR analysis and participated in phenotyping and statistical data analysis, FW designed and revised genotyping of the mutant collection. MB designed and revised the flower phenotype analysis. EC participated in the phenotyping assays and conducted flowering time experiments. KH designed the project as a whole, approved the data and wrote the manuscript.

## Funding

This project was supported by The Academy of Finland (Suomen Akatemia #283138, #256094, #250972) and Becas Chile from Comisión Nacional de Investigación Científica y Tecnológica (CONICYT) Chile.

### Conflict of interest statement

The authors declare that the research was conducted in the absence of any commercial or financial relationships that could be construed as a potential conflict of interest.
